# Adrenergic signaling during parasitic infections

**DOI:** 10.3389/fimmu.2025.1691005

**Published:** 2026-01-26

**Authors:** Patrycja Gardias, Piotr Bąska

**Affiliations:** 1 Department of Pathophysiology and Immunology, National Institute of Geriatrics, Rheumatology, and Rehabilitation (NIGRiR), Warsaw, Poland; 2 Division of Pharmacology and Toxicology, Department of Preclinical Sciences, Institute of Veterinary Medicine, Warsaw University of Life Sciences, Warsaw, Poland; 3 Laboratory of Parasitology, Military Institute of Hygiene and Epidemiology, Warsaw, Poland

**Keywords:** adrenaline, adrenoreceptors (ARs), central nervous system, helminths, neuroinflammation, noradrenaline, parasites, protozoa

## Abstract

Adrenergic signaling plays a critical role in modulating immune and physiological responses during parasitic infections. Catecholamines such as adrenaline and noradrenaline interact with adrenergic receptors (ARs) to regulate immune cell activity, inflammation, and systemic processes. This review highlights the involvement of adrenergic pathways in infections caused by protozoa (*Trypanosoma* spp., *Plasmodium* spp., *Toxoplasma gondii, Leishmania* spp.) and helminths (cestodes, nematodes, and flukes). Central nervous system invasion by parasites is associated with neurodegeneration, mediated by immune and adrenergic mechanisms. Dysregulation of adrenergic signaling can exacerbate infection outcomes or contribute to immune-mediated tissue damage. Understanding these mechanisms provides insights into the potential of targeting adrenergic pathways to improve therapeutic strategies and manage parasitic infections effectively.

## 1 Introduction

**Figure 1:**
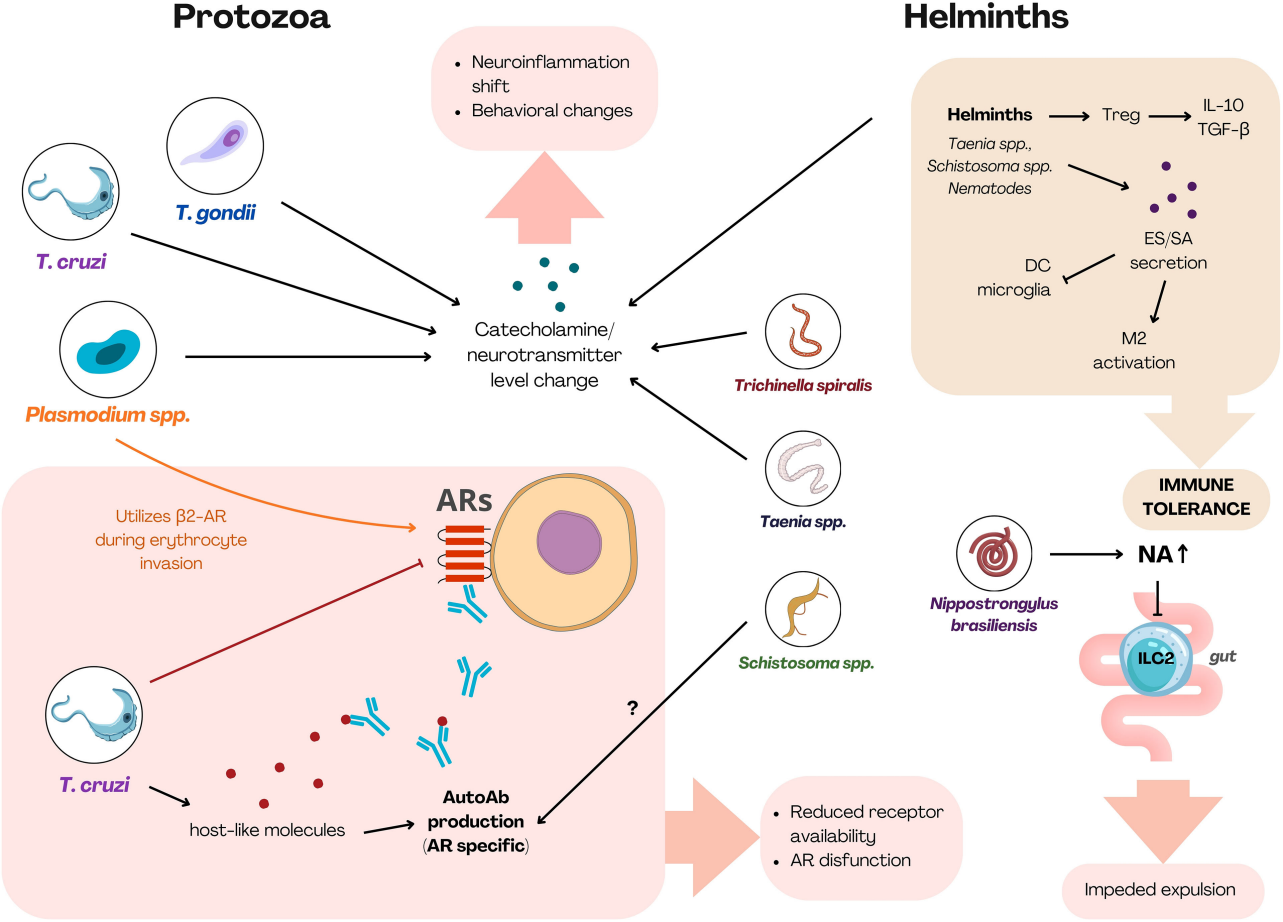
Summary figure. The figure outlines the major mechanisms by which protozoa and helminths modulate host immunity through adrenergic pathways. Protozoa primarily affect adrenergic signaling by altering receptor expression (*T. cruzi*), inducing the production of AR-specific autoantibodies (*T. cruzi*), manipulating neurotransmitter levels (*T. cruzi, T. gondii, Plasmodium* spp.), and exploiting AR-dependent entry or survival pathways (*Plasmodium* spp.). Helminths predominantly drive strong and general regulatory immunity, which includes suppressing immune activation via ES products. Locally helminths modulate ILC2 function through β_2_-AR signaling (*N. brasiliensis* – gut), alter catecholamine/neurotransmitter levels (*T.* sp*iralis*, *Taenia* spp., *Schistosoma* spp.), and in case of *Schistosoma* spp. induce AR-targeting autoantibodies. Shared mechanisms utilized by both protozoa and helminths include disruption of adrenergic signaling, skewing toward suppressive immunity, neurotransmitter dysregulation, and the emergence of functional autoantibodies. ARs, adrenoreceptors; Treg, regulatory T cell; ES, excretory-secretory products; SA, surface antigens; DC, dendritic cell; NA, noradrenaline; AutoAb, autoantibody; ILC2, innate lymphoid cell-2. [Created using Canva Pro (licensed version) based on literature discussed in this work].

Parasitic infections remain a major global health burden, with high prevalence particularly in tropical and subtropical regions (1). For example, soil-transmitted helminthiasis alone affects over 1.5 billion people, especially in developing regions (2). Despite their diversity, parasites share a defining biological feature: intricate life cycles that hinder both diagnostics and treatment, allowing them to persist and spread across populations. Chronic infections and complex life stages, such as hypnozoites in *Plasmodium vivax*, make eradication difficult (3). Although parasites differ widely in taxonomy, morphology, tissue tropism, and host range, they converge on several conserved strategies that enable survival within the host environment. Among these shared strategies is the ability to communicate with and manipulate the host’s immune system – and, through immunological pathways, to influence the nervous system as well (4–6). Parasitic helminths, for instance, secrete a broad repertoire of effector molecules (Excretory-Secretory products - ES) that actively modulate host immunity (7). Growing evidence suggests that parasites can alter adrenergic signaling, one of the key regulatory pathways in the body. Immune cells express adrenergic receptors – ARs (e.g., β_2_-AR), and their activation can shift the immune response (8–10). However, much remains to be discovered about how these pathways function in different parasitic infections. This review aims to highlight many of the studied adrenergic signaling pathways through which parasites may modulate host immunity, reshape host behavior, or influence neurotransmitter levels. A concise, cross-sectional summary of the key mechanisms discussed throughout the entire review is presented in **Figure 1** – Summary Figure.

### 1.1 Catecholamines

Adrenaline (epinephrine) and noradrenaline (norepinephrine), along with dopamine, belong to the catecholamine class compounds renowned for their critical role in stress response and the central “fight or flight” mechanism (11). They belong to adrenal medulla hormones, though certain sympathetic neurons and locus coeruleus in the central nervous system (CNS) can release both adrenaline and noradrenaline (12). These mediators act on effector cells through ARs, mediating various physiological effects, *e.g.*, changes in vascular tone, heart function, metabolic processes, thermoregulation, and the functions of the digestive system (13, 14).

**Figure 2:**
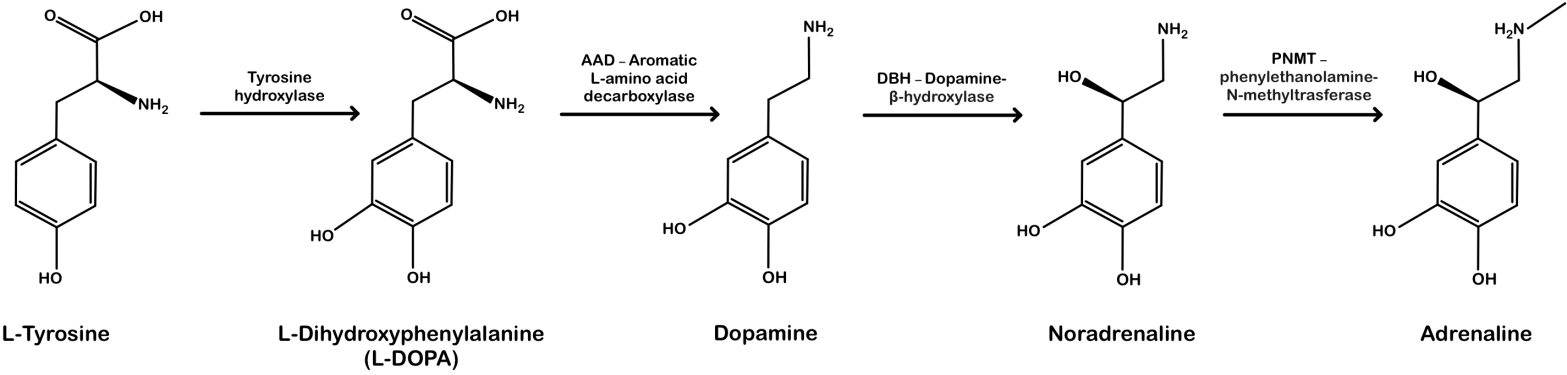
Noradrenaline and adrenaline biosynthesis pathway. Enzymes involved in the biosynthesis of each compound are shown above the arrows. [Created manually based on publicly available information on the chemical structures of compounds and the enzymes involved in their biosynthesis].

Catecholamines’ main structural feature is a benzene ring with two hydroxyl groups attached to adjacent carbon atoms (the catechol group) and an ethylamine side chain; the compounds differ in the presence of one methyl group (**Figure 2**) (15). Their biosynthesis follows a sequential pathway beginning with the amino acid L-tyrosine, which is converted into L-DOPA, followed by the synthesis of dopamine, noradrenaline, and finally adrenaline (15). Noradrenaline biosynthesis is catalyzed by dopamine β-hydroxylase, while adrenaline is produced from noradrenaline via phenylethanolamine N-methyltransferase (**Figure 2**).

Adrenaline and noradrenaline act as ligands for ARs, which belong to G protein-coupled receptors (GPCRs) (16), expressed on various cell types, including immune cells (17–20). In humans, nine AR subtypes are described: α_1A_, α_1B_, α_1D_, α_2A_, α_2B_, α_2C_, β_1_, β_2_, and β_3_, each with unique properties, specificity, and tissue distribution (21). Agonist binding at the orthosteric site of the receptor induces a salt bridge formation with the ligand’s amino group, stabilized by additional hydrogen bonds and π-π interactions (21). This leads to the activation of intracellular signaling pathways via specific G-proteins coupled to particular receptors (22). For instance, α_1_-ARs are coupled with G_αq_ protein, which activates phospholipase C, leading to PIP2 (phosphatidylinositol 4,5-bisphosphate) hydrolysis into IP3 (inositol 1,4,5-triphosphate) and DAG (diacylglycerol) (23). In contrast, α_2_-ARs are coupled with G_αi_, suppressing adenylyl cyclase activity and decreasing intracellular cAMP (cyclic adenosine monophosphate) levels, whereas β-ARs, coupled with G_αs_, stimulate adenylyl cyclase activity, resulting in cAMP increase (23). Moreover, ARs may be activated by both adrenaline and noradrenaline in different manners, as each receptor has a specific affinity for adrenaline or noradrenaline (**Table 1**) (24).

**Table 1:** AR subtypes and their preferred ligands.

AR subtype	Preferred ligand
α_1_-AR	N>A
α_2_-AR	N<A
β_1_-AR	N>A
β_2_-AR	N<<A
β_3_-AR	N=A

α_1_-ARs preferentially bind noradrenaline, contrary to α_2_-ARs. β_1_-ARs bind noradrenaline with higher affinity than adrenaline. β_2_-ARs have a higher affinity for adrenaline than noradrenaline, whereas β_3_-ARs bind both agonists with equal potency. **N** – noradrenaline; **A** – adrenaline.

## 2 The role of ARs in the neuroimmune response

Although classically immune cell interactions are regulated by cytokines and chemokines, in recent years, molecular mechanisms of the neuroimmune response have attracted more attention. The nervous system responds to a variety of both external and internal stimuli, and due to its function, it is embedded in every body tissue, collecting and processing information about the organs’ current states (25). It is evident that neurons, in cooperation with the immune system, can sense the pathogen presence and other homeostatic deviations, regulating the immune response against the intruders (26). Afferent neurons sensing ongoing inflammation or injury transmit signals through the vagus nerve to the brainstem, where the information is processed (27). This leads to the activation of the efferent vagus nerve-mediated cholinergic anti-inflammatory pathway, reducing the release of pro-inflammatory cytokines. This centrally integrated neuroimmune mechanism is known as the inflammatory reflex (27). Moreover, the interplay between neurons and immune cells is a substantial component of homeostasis regulation; neurons are also involved in inflammation modulation, and they thus participate in coordinating immune response during infections (28–31), pathological conditions (*i.e.*, neurodegeneration and autoimmunity) (32, 33), pain sensation, and injuries (34–36). Neurons and immune cells communicate through physical contact and numerous released mediators affecting specific receptors on both cell populations (37–40). The released molecules belong to both cytokines and neuromediators; therefore, they can alter neuronal signaling, pain sensitivity (41), and neuronal physiology, as well as modulate both innate and adaptive immune responses (42). Moreover, the interplay between neurons and immune system is usually tissue-specific, with specific molecule profiles and hallmarks, like in lungs, intestine, or central nervous system (CNS) (43–45). In the following sections, we discuss adrenergic signaling – a key pathway in neuroinflammation – and its role in modulating neuroinflammatory responses within peripheral immune cells, the CNS, and the intestine.

### 2.1 Peripheral immune cells

The nervous system innervates both primary and secondary lymphoid organs, participating in orchestrating the immune response (46, 47). Neurons located in these organs belong to the sympathetic nervous system and participate in immune regulation (48), and immune cell migration (49), which are most likely mediated by adrenergic signaling. Substantial innervation by sympathetic nerve fibers was also described in the spleen; however, depending on the species, the innervation pattern differs significantly, with more restricted innervation in humans compared to rodents (50). Noradrenaline release in lymphoid organs can affect immune system activity, including cell differentiation, proliferation, and regulation of innate and adaptive responses (51–54). In the murine spleen, an α_2_-AR antagonist increases noradrenaline release, which further decreases the production of inflammation mediators, such as TNF-α, IL-6, and IL-10, involved in the functioning of macrophages, T cells, and dendritic cells (DCs) (55). Simultaneous inhibition of α_2_-AR and increased noradrenaline release additionally corroborate the autoregulatory mechanism of α_2_-AR (55). Adrenergic receptors are present on various immune cell types (**Table 2**), with the most abundantly represented subtype being β_2_-AR; for instance T cells and B cells express β_2_-AR almost exclusively (56). However, it is now clear that immune cells also express other AR subtypes, including some of the α_1_-AR family. Yet, AR expression profiles are diversified and depend on cell type, location, or surrounding environment (57). Interestingly, PBMCs lack α_1_-ARs of any subtype (58); nevertheless, α_1_-AR expression can be elicited *in vitro* by stimulation with phytohemagglutinin mitogen (PHA) and lipopolysaccharide (LPS) (59). Additionally, IL-1β and TNF-α stimulation of the THP-1 monocytes results in α_1d_-AR and α_1a_-AR expression, while exposure to IL-6 and IL-8 has no effect (60). Concurrently, increased expression of α_1b_-AR and α_1d_-AR (but not α_1A_-AR) is elicited by a β_2_-AR agonist (terbutaline), suggesting cross-regulation of α_1_-AR expression in PBMC (61). In innate immune cells, α_1_-AR downstream signaling leads to the exacerbation of inflammation, as shown by the use of both their agonists and antagonists. Treatment with agonists increased production of pro-inflammatory cytokines (62), whereas antagonists attenuated inflammation (63). The role of adrenergic receptors in immune response modulation was also confirmed in mouse laboratory models, where bone marrow cells from *Adrb1^-/-^* and *Adrb2^-/-^* mice were transferred to previously irradiated C57BL/6J mice (64). The study showed a decreased number of circulating T cells, macrophages, and neutrophils, along with modifications in the transcription profile, including genes involved in the recruitment, activation, and cytokine release of immune cells (64). Moreover, *Adrb2* deletion in mice also causes severe loss of IL-10 and increased TNF levels in response to LPS, indicating profound anti-inflammatory properties of *Adrb2* (20). This may correlate, in part, with findings from another study, where authors showed that macrophage polarization toward M2 phenotype and reduction of cytokine production might be associated with β_2_-AR (65), which is generally considered an inhibitor of immune cell activation (66–68). Slota et al. (2015) demonstrated that human CD8^+^ memory T cells exposed to noradrenaline *in vitro* show altered cytokine expression and reduced activation-induced proliferation (52). Although many studies confirm inhibitory properties of noradrenaline, in some cases, it facilitates the initiation of the immune response. It was shown that noradrenaline promotes IL-12-mediated differentiation of naive CD4+ T cells into the Th1 phenotype, leading to increased IFN-γ levels (69). Moreover, noradrenaline enhances IgG1 and IgE production by B cells previously exposed to Th2 cells (70). However, observations provided by Melmon et al. (1987) indicate that plasma cells may reduce antibody production upon exposure to β-AR specific amines, including noradrenaline (71). Latest findings show that noradrenaline directly modulates B-cell function; it enhances antibody affinity through β_2_-AR activation (72) and regulates neuroimmune axis in germinal centers (73). B cells also synthesize catecholamines, and AR-mediated signaling increase their regulatory function (Bregs) (74). These findings underscore the importance of systemic adrenergic signaling and its pivotal role in immune system modulation.

**Table 2:** Distribution of ARs across human immune cells.

Innate immunity	α_1A_	α_1B_	α_1D_	α_2A_	α_2B_	α_2C_	β_1_	β_2_	β_3_
Monocytes	++	++	++	?	?	?	[+]	+	?
Macrophages	+	?	+	+	+	+	[+]	+	+
Neutrophils*	+	+	+	+	–	+	+	+	+
Eosinophils	?	?	?	–	?	?	?	+	?
Basophils	?	?	?	[+]	[+]	[+]	?	+	?
Mast cells	+	+	+	?	?	?	+	+	–
NK cells	[+]	[+]	[+]	[+]	[+]	[+]	–	+	?
DCs	?	?	?	?	?	?	+	+	?
Microglia	?	?	?	[+]	?	?	[+]	+	?
Astrocytes	?	[+]	?	[+]	?	?	[+]	+	?
ILCs	?	?	?	?	?	?	?	+	?
Adaptive immunity	α_1A_	α_1B_	α_1D_	α_2A_	α_2B_	α_2C_	β_1_	β_2_	β_3_
Lymphocytes	++	++	++	[+]	[+]	[+]	**□**	+	**□**

“+” - present; “[+]” - possibly present; “++” - present upon activation; “**□**” - possibly present upon activation; “–” - absent; “?” - data not available.

*Neutrophil: β_3_>β_2_>α_1A_>α_1B_~α_2A_~β_1_=α_1D_=α_2C_.

### 2.2 Central nervous system

Noradrenaline, present in the CNS, primarily originates from the locus coeruleus and is a substantial mediator participating in arousal, fear, anxiety, and other stress-mediated responses (75). Although the brain is protected by the blood-brain barrier from detrimental inflammation (76), peripheral immune cells have an ability to infiltrate the CNS. Moreover, microglia cells abundant in the brain show immune potential (77, 78). They are involved in regulating brain development, neuron regeneration, pathogen elimination, and brain “cleaning”, which includes synapse pruning, elimination of dead and superfluous cells, as well as removal of various molecule aggregates and potentially dangerous antigens (79–81). Similarly to peripheral macrophages, microglia can be classically and alternatively activated and classified as M1 and M2, respectively (82). Classic activation of microglia towards the pro-inflammatory phenotype (M1) is primarily induced by IFN-γ and TLR signaling (mainly TLR4), leading to TNF-α, IL-6, IL-1, IL-1β and chemokines secretion, which promote inflammation (83, 84). In contrast, the M2 phenotype, which is mostly anti-inflammatory, contributes to neuronal protection (85) and reduction of inflammation through TGF-β, arginase 1 (Arg-1), and various growth factors (86). Another finding indicating anti-inflammatory properties of M2 was provided during IL-10 deficiency, which caused conversion of M2 microglia into M1 phenotype and loss of M2-specific markers (87). Microglia express a significant number of ARs, both β- and α-ARs (88), making them particularly sensitive to the slightest alterations in noradrenaline levels in the CNS (89). Lower catecholamine levels, observed during CNS disorders, infection, or even anesthetics, can activate microglia and lead to pro-inflammatory activity (90, 91), suggesting that higher levels of catecholamines may produce the opposite, anti-inflammatory effect in the CNS. Concurrently, noradrenaline secreted by neuronal cells inhibits microglial IL-1β, which is partially responsible for tissue damage, thus confirming noradrenaline’s protective properties (92). Depending on the activation state (resting or activated), noradrenaline can affect their functions through different ARs, achieving similar results (88). Noradrenaline causes retraction of microglial cellular extensions in both resting and activated microglia. In resting cells, this effect is mediated by β_2_-ARs, while in activated microglia, it involves α_2A_-ARs (88). However, β-adrenergic signaling potentially plays a role in anxiety-resembling behavior associated with social defeat and other stressors mediated by microglia activation, leading to increased expression of IL-1β and other inflammation factors (93). Mice exposed to repeated social defeat and treated with propranolol (a β_2_-AR antagonist) restored IL-1β levels and showed reduced social-defeat-induced anxiety (93).

In pathological conditions such as injury, monocytes can infiltrate the CNS (94). There, chemokines attract M1 macrophages followed by latter M2 infiltration. M1 macrophages migrating to the CNS are derived from monocytes entering the spinal cord through adjacent spinal leptomeninges in a CCL2-dependent manner, whereas M2 macrophages originate from monocytes migrating through the brain-ventricular choroid plexus, indicating that M2 macrophages can travel longer distances than M1 (94, 95). This was confirmed by showing that M2 macrophages, in response to neuroinflammation-related chemokines (CCL2, CCL5, CXCL10, CXCL12 and C1q), have the strongest capacity for long-distance migration, compared to M0 and M1 macrophages, what is assumed to be associated with differences in morphology and cytoskeletal arrangement between the M1 and M2 phenotype (96). Apart from chemokines, GM-CSF can also enhance macrophage recruitment and migration through the blood-brain-barrier, while concurrently activating microglia (97).

Astrocytes, cells present in the grey matter, play a key role in regulating the blood-brain-barrier integrity; they support neurons, maintaining the CNS homeostasis (pH, ion levels, neurotransmitter recycling) (98) and release numerous compounds, including prostaglandins, arachidonic acid, and nitric oxide (NO) (99–101). They may also release cytokines such as TNF-α, IL-6, and IL-1, mediating neuroinflammation in response to various homeostatic disturbances (102, 103). Human astrocytes predominantly express β_2_-ARs (104), whose agonists appear to impact expression of the TNF-activated genes, such as: IL-6, CXCL2, VCAM1, ICAM1 (105). Clenbuterol (a β_2_-AR agonist) administered with TNF increases the CD4^-^ CD8^-^ T cell population, implying that astrocytes participate in neuroimmune modulation (105).

During the course of neurodegenerative diseases, adrenergic signaling is crucial for neurons’ activity and maintaining their cognitive function. Previous studies indicated that the α_1_-AR antagonist prazosin facilitates anti-inflammatory cytokine production and improves cognitive function in APP23 mice (a model of Alzheimer’s disease (AD)) (106) and reduces aggression and excitability in AD patients (107). Conversely, α_2_-AR blockers (yohimbine, mesedin) increase noradrenaline levels, leading to elevated IL-10, known for its neuroprotective and neurogenic properties (108, 109). It was also shown that these blockers can increase survival rates in animals, reduce anxiety, and improve memory and cerebral blood flow in a focal ischemia model (109). Additionally, mesedin promotes neuron survival and development in primary astroglial cultures from C57BL/6 and 3×Tg-AD mice (109). β_2_-AR signaling in astrocytes may also play a role in neurodegenerative diseases such as multiple sclerosis (MS) and AD (110, 111). For instance, patients with MS have significantly reduced astrocytic β_2_-AR expression in both MS plaques and in “normal appearing white matter” (NAWM) (112). Similarly, in dogs with chronic distemper encephalitis, astrocytes in NAWM and demyelinated lesions lack β_2_-ARs (113). In AD pathophysiology, abnormal β-amyloid deposition is central to disease progression and subsequent dementia (114), and a substantial body of research have shown interplay between β-amyloid and adrenergic signaling (115–120). β-amyloid accumulations burden locus coeruleus neurons and cause their degeneration (121, 122). In rats, β_2_-adrenergic activation was shown to prevent β-amyloid-induced inhibition of long-term potentiation (LTP) (118). Additionally, in murine astrocytes, β-amyloid increases cAMP and apolipoprotein-E (apoE) levels, which are reduced by β-AR antagonists (especially β_2_-AR), suggesting that β-AR alienation can mitigate β-amyloid cytotoxicity (119). In corroboration with these finding, in rats, isoproterenol (β-AR agonist) and noradrenaline were shown to increase amyloid precursor protein APP mRNA and holoprotein levels. This effect was blocked by propranolol (120). These studies suggest that adrenergic signaling may play an important role in neurodegenerative processes or, alternatively, may facilitate neurons’ survival and CNS homeostasis.

### 2.3 Intestine

The network of neurons embedded in the abdomen area is classified, depending on their location, as either part of the intrinsic – neurons innervate the walls of gastrointestinal tract – or extrinsic enteric nervous system (ENS) (123). Extrinsic sympathetic and parasympathetic neurons are strictly controlled by the brainstem and spinal cord and impact intestinal motility (124). Sympathetic extrinsic neurons respond to catecholamines via α and β-ARs, through which inhibitory signals are transmitted to both the small and large intestine (125). On the other hand, the vagus nerve serves as the source of parasympathetic neurons, whose role is to mediate cholinergic signaling, thereby regulating overall intestinal physiology, including peristaltic movements and digestion (126). Intrinsic enteric neurons are located within all the intestinal tissue layers and consist of the myenteric and submucosal plexi (124, 127). Both the nervous system and immune system have developed mechanisms enabling pathogen attenuation and/or eradication during the infection. Neurons continuously exchange molecular signals with various populations of immune cells (innate lymphoid cells (ILCs), macrophages, mast cells, and lymphocytes) residing in the gut and surrounding tissue (128). The significant role of muscularis macrophages (MM) is to support the survival of intrinsic enteric-associated neurons during infection through the Arg-1-polyamine axis (129); polyamine production is stimulated by activation of the β_2_-AR in macrophages, which is triggered by catecholamines such as noradrenaline released by sympathetic neurons projecting to the gut (129). Interestingly, β-AR activation in human monocyte-like cells is generally associated with anti-inflammatory effects (130–132). Additionally, Gabanyi et al. have thoroughly examined the influence of β_2_-AR on gut MM. During the bacterial infection, adrenergic signaling mediated by rapid sympathetic neuron noradrenaline release promotes an alternative activation in gut-residing macrophages (44). Furthermore, the expression of *Arg1* gene, associated with the M2 macrophage phenotype, neuron development and anti-apoptotic effects, was upregulated in peritoneal macrophages as a result of stimulation with noradrenaline and salbutamol (β_2_-AR agonist) (44). Besides resident macrophages, neurons also interplay with *Adrb2*-expressing ILC2s, impeding type 2 response. In intestinal mucosa, *Adrb2* deletion leads to an increased acute type 2 response, while *Adrb2* activation (clenbuterol) notably inhibits ILC function in wild-type C57BL/6 (B6) mice (31). Other substantial population of immune cells in the gut are mast cells, which release a range of compounds including vasoactive and inflammatory mediators, cytokines, and neurotransmitters (133). These molecules affect adjacent neurons leading to visceral pain, and *vice versa* sympathetic and parasympathetic neurons can regulate mast cell degranulation through the neurotransmitters they release (134, 135). These interactions may indeed involve adrenergic signaling, as lung mast cells, which are associated with exacerbation of asthma symptoms, express β_2_-AR (136); importantly, β_2_-AR agonists used as bronchodilators are among the most effective treatments for limiting mast cell degranulation (137–139). At this stage, further investigation into adrenergic signaling within the gut environment is required to gain a deeper understanding of the ongoing processes.

## 3 Adrenergic signaling during parasitic infections

### 3.1 Protozoa

Protozoan parasites are unicellular organisms responsible for the development of severe diseases that affect millions of people worldwide; globally, intestinal protozoan infections impact up to 3.5 billion individuals (140). Among the most challenging protozoan-borne diseases are Chagas disease (*Trypanosoma cruzi*), African trypanosomiasis (*Trypanosoma brucei*), malaria (*Plasmodium falciparum*), toxoplasmosis (*Toxoplasma gondii*), and leishmaniasis (*Leishmania* spp.). These protozoa are obligatory intracellular parasites with exceptionally complexed life cycles and are capable of invading many different organs and types of nucleated cells (141, 142). The ability to invade various cells facilitates parasite spreading and settling within the host tissues, which makes the infection problematic to diagnose and treat (143, 144). Considering the parasites’ ability to modulate the host’s immune system in order to ameliorate its response, it is noteworthy that these mechanisms also involve adrenergic modulation. For better understanding of these molecular interactions, in the following sections we aim to review the existing knowledge regarding this issue during the course of *Trypanosoma* spp., *Plasmodium* spp.*, Toxoplasma gondii* and *Leishmania* spp. infections. A summary of the AR agonists and antagonists used in the studies described in this manuscript, in the context of parasitic infections, is presented in **Table 3**.

**Table 3:** Summary of adrenergic receptor (AR) agonists and antagonists and their reported effects in protozoan and helminth infections.

Compound	Target receptor	Agonist/Antagonist	Reported effect	Associated parasite model	Citation
PROTOZOA					
Adrenaline	Various ARs	Natural agonist	- Inhibits *L. donovani* attachment to macrophages; - Reduces *Leishmania* growth *in vitro*	*Leishmania donovani*	(257) (258)
Noradrenaline	Various ARs	Natural agonist	Inhibits *L. donovani* attachment to macrophages	*Leishmania donovani*,	(257)
Isoproterenol; L-isoproterenol	β-AR	Agonist	- Reduces *Leishmania* growth *in vitro*; - Reduces % of *T. cruzi*-infected macrophages and reduce parasites per cell	*Leishmania donovani;* *Trypanosoma cruzi*	(258) (153)
Propranolol	β-AR	Antagonist	- Reduces *L. mexicana* lesion size and parasite load; - Reduces *L. donovani* growth *in vitro*	*Leishmania mexicana*, *Leishmania donovani*	(264) (258)
Atenolol	β_1_-AR	Antagonist	Reduces hyperalgesia and TNF-α in *L. major* infection	*Leishmania major*	(265)
Clonidine	α_2_-AR	Agonist	Decreases number of *T. gondii-infected* macrophages (direct effect on parasite)	*Toxoplasma gondii*	(250)
Guanabenz	α_2_-AR	Agonist	Reduces neuroinflammation in mice with latent toxoplasmosis; dampens inflammatory cytokine release	*Toxoplasma gondii*	(247, 248)
Salmeterol; Phenylpherine	β_2_-AR α_1_-AR	Agonists	Inhibit *Plasmodium*-specific CD8+ T cell proliferation, thus facilitates invasion; delay CNS invasion	*Plasmodium berghei*	(195)
L-phenylephrine	α_1_-AR	Agonist	Enhances the interaction of T. cruzi with host cells	*Trypanosoma cruzi*	(153)
HELMINTHS					
Noradrenaline	β_2_-AR (ILC2)	Natural agonist	Diminishes ILC2-mediated type 2 response; indirectly impedes parasite expulsion	*Nippostrongylus brasiliensis*	(31)
β-AR blockers/β_2_-AR inhibition (functional importance, not specific drug name given)	β_2_-AR	Antagonists (general reference)	β_2_-AR deficiency reduces M2 macrophage formation, mitigates liver fibrosis and hepatobiliary damage	*Clonorchis sinensis*	(348)

The table was prepared based on the literature reviewed in this manuscript.

#### 3.1.1 Trypanosoma spp.

**Figure 3:**
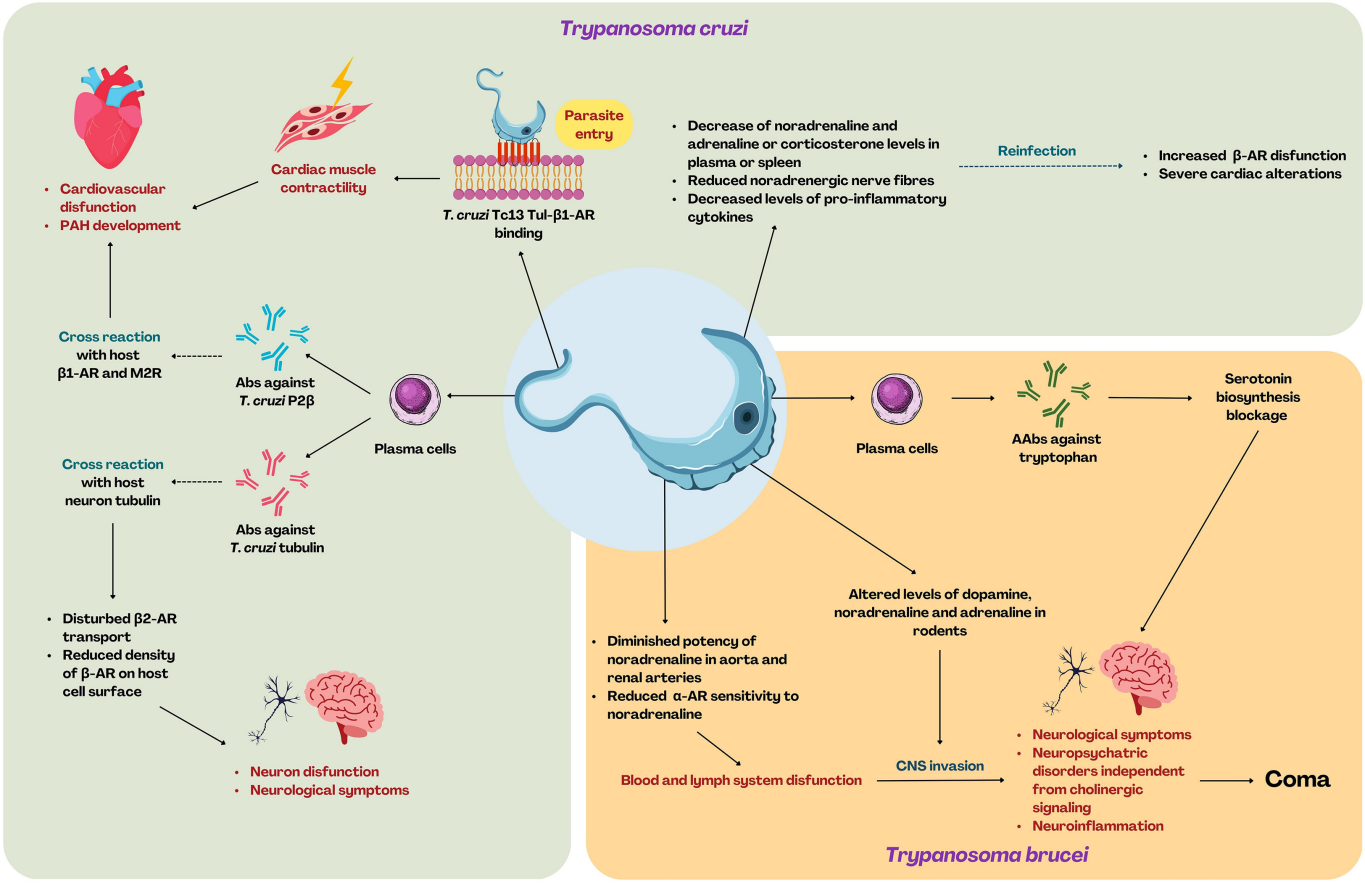
The impact of *T. cruzi* and *T. brucei* infection on β-AR-mediated neuroimmune response and sequential manifestations. *T. cruzi* causes cardiovascular dysfunction and pulmonary arterial hypertension (PAH) through the stimulation of 1-AR, as well as by promoting the production of autoantibodies cross-reacting with β_1_-AR and M2R. *T. cruzi*-mediated production of AAbs also impacts β2-ARs, causing neurological symptoms and dysfunction of the neurons. Decreased levels of neurotransmitters such as noradrenaline and adrenaline, and decreased levels of pro-inflammatory cytokines, favor exacerbation of the host condition during reinfection. *T. brucei* contributes to alteration within neurotransmitter secretion levels, thus impeding the α-AR sensitivity. *T. brucei* promotes B cell differentiation toward plasma cells and the production of the AAbs against tryptophan, causing the inhibition of serotonin biosynthesis. The above-mentioned complications lead to blood and lymph system dysfunction, facilitating the migration to the CNS and subsequent neuroinflammation, neurological and neuropsychiatric symptoms, and eventually coma. [Created using Canva Pro (licensed version) based on literature discussed in this work].

Chagas disease, caused by *Trypanosoma cruzi*, is a severe disorder manifested by various cardiological and gastrointestinal symptoms (145). The parasite is transmitted via the feces of “kissing bugs” (hematophagous triatomine bugs), but it can also be transmitted directly through blood transfusion, orally, or congenitally (146). The most fatal and frequent complication associated with Chagas disease is cardiac involvement, resulting in cardiomyopathy, followed by refractory heart failure and thromboembolism during chronic phase, leading to sudden death (147). Cardiomyopathy is most likely driven by dysregulation of the adrenergic signaling in the cardiovascular system. *T. cruzi* infection leads to reduced density of β-AR on the surface of host cardiac cells (148–150) and decreased levels of adrenaline and noradrenaline in plasma (151). Additionally, in the blood of mice, an increased level of corticosterone was observed, and a reduced level of noradrenaline was found in the spleen (152). It appears to be related to the disruption of cardiovascular system functioning, reduced noradrenergic nerve fibers or reduced pro-inflammatory cytokine release (152). Moreover, even more severe cardiac alterations and increased cardiac β-AR dysfunction are observed during reinfection (148). In 1998 Connely et al. showed that AR agonists reduce the percentage of *T. cruzi* infected macrophages and decrease an average number of parasites per cell, suggesting that AR agonists may also modulate infectious potential of *T. cruzi in vivo* (153). Although the mechanism of cardiological disruption and cardiomyopathy development is still not clear, it appears that it might be associated directly with *T. cruzi* activity or the immune response raised against the parasite (**Figure 3**). *T. cruzi* interacts with host cells (direct cell-to-cell contact) through its Tc13 Tul surface molecule, which binds to β_1_-AR on the host cell surface, probably allowing entry to the host cell (154). Tc13 Tul also acts as an adrenergic agonist, promoting intracellular signaling, which results in constant stimulation of cardiac muscle contractility (154). Other mechanisms of cardiomyopathy development associated with AR involves *T. cruzi* molecular mimicry and cross-reactivity of antibodies against its antigens. In the blood of humans suffering from Chagas disease, autoantibodies (AAb) recognizing β_1_-AR and muscarinic M2 receptor (M2R) were detected (155). These antibodies are primarily targeted against the C-terminal region of the ribosomal P2β protein of *T. cruzi* (156). However, due to structural similarities, upon binding to β_1_-AR and M2R, they act as agonists and may lead to tissue and organ damage, ultimately resulting in cardiovascular dysfunction (157). One such AAb exhibiting these properties is murine Ab 17.2 against *T. cruzi* P2β, which acts as a β_1_-AR agonist both in humans and rats (158). Dogs, on the other hand, exhibit additional AAbs recognizing β_2_-AR during the infection. Although these AAbs generally cause autoimmune-like symptoms in cardiovascular system, their severity may depend on the *T. cruzi* strain (159). Silvina Lo Presti et al. (2006) showed that cardiac sensitivity to noradrenaline was decreased in mice, with a more pronounced reduction for the *T. cruzi* SGO Z12 strain than for the *T. cruzi* Tulahuen strain (151).

Less frequently, patients suffering from Chagas disease may develop neurological symptoms, involving meningoencephalitis, headaches, tumor-like forms, lethargy, and mood changes in the acute phase (160, 161) or neuronal damage and autonomic system dysfunction during the chronic phase (162). Recently, A2R1 Ab against *T. cruzi* tubulin was proved to react also with mammalian neuronal tubulin (163), probably leading to neuronal dysfunction; however, the precise mechanism remains elusive. Nevertheless, A2R1 might be associated with impaired function of β_2_-AR and reduced density of the receptor on the cell surface. Duvernay et al. (2011) showed that tubulin is essential for β_2_-AR transport from the rough endoplasmic reticulum (RER) and disruption of this pathway leads to accumulation of the receptor in RER, abrogating cell functioning (164). In vascular smooth muscle cells, noradrenaline acting through adrenergic receptors can cause reduced expression of cytoskeletal protein (*i.e.*, sm-α-actin, β-tubulin and desmins) (165). These findings may indicate a potential relation between β_2_-AR-tubulin interaction and neurological and cardiological symptoms in Chagas disease, which opens a new avenue of investigation.

*Trypanosoma brucei*, causing sleeping sickness (Human African Trypanosomiasis - HAT) is transmitted by a specific vector tsetse fly (166). There are two parasite subspecies capable of infecting humans: *T. b. gambiense* and *T. b. rhodesiense* (167). Sleeping sickness is often associated with the presence of two stages: the hemolymphatic stage, in which trypanosome invasion is restricted to blood and lymph system, and the meningo-encephalitic stage, which is defined by CNS involvement (168). It is suspected that some of the occurring symptoms, especially those manifesting CNS dysfunction, may be correlated to dysregulation of adrenergic signaling (**Figure 3**). Unfortunately, there is a limited number of studies examining this aspect, and the data usually come from animal models. Similarly to *T. cruzi*, *T. brucei* infection was proven to alter ARs properties. Diminished potency of noradrenaline in aorta and renal arteries (in infected rats) and reduced sensitivity of α-AR to noradrenaline (in infected rabbits) have been observed (169). Since neurotransmitters play a crucial role in brain activity, sleep, and wakefulness (170), neuropsychiatric disorders associated with HAT were suggested to be the result of monoaminergic alterations in the brain (171, 172). In *T. b. brucei*-infected mice, decreased levels of dopamine or noradrenaline were observed (171). In addition, the authors emphasized that the cholinergic signaling system is most likely not affected by *T. cruzi* infection (171). In contrast, studies provided by Stibbs (1984a, 1984b, 1987) referring to *T. b. gambiense* infection in animal models showed unchanged dopamine and noradrenaline levels in infected voles and unaffected dopamine levels in mice, while infected rabbits and rats showed increased levels of dopamine; in addition, decreased levels of serotonin in each case were observed (172–174). As previously mentioned, *Trypanosoma cruzi* infection is linked to the Ab production against parasite’s antigens, which cross-react with the host antigens. Similarly, in HAT patients’ sera, the AAbs against tryptophan epitopes were found (175, 176). Since tryptophan is a precursor to serotonin (177), it may impact serotonin biosynthesis and restrict its bioavailability. This outlines the importance of neurotransmitters in disease development during *T. brucei* infection, however, the limited and dated evidence is certainly not sufficient for understanding the complex mechanism of *T. brucei* infection, therefore further studies are required to comprehend these dynamics.

#### 3.1.2 Plasmodium spp.

Malaria, caused by protozoan parasite *Plasmodium* spp., is a life-threatening, tropical disease occurring mainly in tropical and subtropical areas (178). *Plasmodium falciparum* is a causative agent of most malaria cases, especially in sub-Saharan Africa; however, in some cases and in various regions, the disease is also associated with *P. malariae*, *P. ovale*, and *P. vivax* infections (179, 180). Malaria manifestations primarily result from hepatocyte and red blood cell (RBC) rupture and dysfunction as well as from the immune response raised against the parasite (181). *Plasmodium* infection is particularly dangerous for children, who are likely to develop severe symptoms manifested by anemia, cerebral malaria, respiratory distress or coma, often leading to sudden death (182). The parasite is easily transmitted through *Anopheles* spp. female mosquitos, which are required for proper *Plasmodium* spp. development and its life cycle closure (183). During mosquito feeding, sporozoites are transferred into the bloodstream. They reach the liver cells and mature inside them into schizonts, eventually causing cell rupture. The released schizonts manage to enter bloodstream once again to infect the RBCs (184). Schizonts then undergo trophozoite development, which then takes one of two paths: immature trophozoites either transform into mature trophozoite and then schizont causing RBCs rupture, or differentiate into gametocytes (sexual erythrocytic stage), which are released and taken by the mosquito with its blood meal (185). Alvares et al. (2014) proved that the parasite interferes with numerous physiological processes on the molecular level: during intraerythrocytic *Plasmodium* stage RBCs effectively adhere to vascular endothelium and non-infected RBCs, causing their entrapment in microcirculation, thus possibly facilitating *Plasmodium* spreading (186). Additionally, the release of extracellular ATP and NO by infected RBCs is enhanced, which causes the vasodilation of microvasculature. Isoproterenol was used in this study as a compound of a cAMP-activating cocktail (along with forskolin, and papaverine) to stimulate ATP release (186). Other studies showed that β_2_-AR antagonists hamper the growth of the parasite (187, 188). Indirect adrenergic signaling seems to play a role also during *Plasmodium* entrance to RBCs. *Plasmodium* schizonts preceding erythrocytic stage can use precursor membrane complex, consisting of CD44 and β_2_-AR (189). Moreover, *Plasmodium*-derived RH5 antigen binds to basigin on RBCs, causing β_2_-AR and G_αs_ protein activation, followed by cAMP and Ca^2+^ level increase, thus enabling the parasite entrance (189). The role of adrenergic signaling seems to be crucial also in hypoglycemia or renal failure, which are common complications of *Plasmodium* infection (190). In *Plasmodium-*infected mice, significantly decreased glucose levels due to adrenalectomy (compared to adrenalectomized control) were observed, which confirms that adrenaline plays a significant role in regulating glucose levels (191). On the other hand, patients showing abnormal water load response followed by increased mean arterial pressure exhibit increased plasma noradrenaline level (192). For instance, during sepsis or LPS-stimulation, noradrenaline enhances IL-10 release, and simultaneously reduces pro-inflammatory cytokine release, which favors infection spreading (132, 193). Therefore, the increase of plasma noradrenaline levels may indicate disease progression in malaria patients. In the context of the above-mentioned findings, catecholamine levels could serve as potential markers for predicting renal failure and hypoglycemia in malaria.

Severe malaria is sometimes associated with cerebral malaria development (194). It was proved that AR agonists (salmeterol and phenylpherine) inhibit *Plasmodium*-specific CD8^+^ T cell proliferation in mice, facilitating *Plasmodium* infection (195). However, the AR agonists also delay CNS invasion, which could be associated with impaired agonist-mediated migration of immune cells (195). Nevertheless, in the invaded brains of *Plasmodium berghei*-infected rodents, the reduced noradrenaline and serotonin levels were observed, which additionally confirms the relation between noradrenaline levels and CNS invasion (196). It is also noteworthy that neurotransmitter level changes in CNS and glutaminergic pathways are often associated with the occurrence of neurological symptoms such as cognitive, behavioral, and motor dysfunctions during malaria infection (196–198). All these data open a question about the use of adrenergic pathways as targets for malaria treatment, which was analyzed by Prabhu et al. (2024) who explored drugs-miRNA interaction *in silico* and suggested antimalaria potential of β-adrenergic drugs, including adrenaline and propranolol (199). These compounds have been used for decades, and their safety and side effects have been scrutinized (200, 201). Therefore, if the hypothesis is confirmed by further experiments, the registration process of β-adrenergic drugs for malaria control will be facilitated and shortened due to number of previously collected data.

#### 3.1.3 Toxoplasma gondii

Toxoplasmosis is one of the most frequent diseases among warm-blooded animals affecting various species of mammals and birds (202). The disease shows the highest prevalence in Latin America, Eastern/Central Europe, as well as the Middle East and the southern part of Africa (203). Its etiological factor is an obligate intracellular parasite *Toxoplasma gondii*, which has a complex life cycle with the presence of various intermediate stages; its definitive host is a cat, which is crucial for sexual reproduction and cycle closure (204). During the life cycle the parasite undertakes three different forms: i) tachyzoites present in body fluids, associated with acute infection; ii) bradyzoites present within tissue cysts, associated with chronic infection; iii) oocysts excreted with cat’s feces (205). Clinical symptoms of the infection are related to specific phases of infection and host immunocompetence. In general, parasite-driven disorders are mediated by mechanisms involving the induction of immune responses, leading to the immunopathologies, disruptions in host physiology, and altered neurotransmitter and hormone levels (206). *Toxoplasma* spp. invades monocytes/macrophages DCs, NK cells, and other leukocytes (207–209), and upon reaching the CNS (Trojan horse mechanism) starts to reside within almost every brain region without particular preference. Here, it forms cysts and remains latent (144). Its presence correlates with the development of neurodegenerative diseases development (Parkinson’s disease, Alzheimer’s disease) (210–212), mental disorders (213, 214), schizophrenia (215), even suicide (216) and traffic accidents (217). Moreover, numerous studies show that in rodents toxoplasmosis leads to an altered behavior associated with adrenergic and dopaminergic signaling modulation (215, 218–221). Additionally, it was suggested that behavioral alterations also depend on host-related factors such as age (222, 223), genetic susceptibility, number/location of cysts (224–226), and the severity of initial acute infection (219, 227). The infection leads to deteriorated learning and locomotor activity (228), altered memory (229), decreased fear, increased sociability (230), and reduced predator odor aversion (226, 231). Noradrenaline and dopamine belong to neuromediators participating in regulation of alertness, arousal, locomotor activity, learning and memory (206, 232) and are presumed to have a significant impact on the aforementioned observations. Surprisingly, despite this hypothesis, the data regarding neurotransmitter levels during the infection are inconsistent. In 1985, Stibbs observed a significant decrease of noradrenaline in acute *T. gondii*-infected animals, while, interestingly, in chronically infected animals an increased level of dopamine was observed (219). These discrepancies were explained by recent studies, which showed differences in neuromediator levels in various brain regions (228). The infected mice had increased levels of noradrenaline and L-DOPA in amygdala; increased noradrenaline, L-DOPA and dopamine in striatum; increased noradrenaline in prefrontal cortex; increased dopamine and decreased noradrenaline, L-DOPA and DOPAC in hippocampus (228). However, the use of a highly virulent *Toxoplasma gondii* strain (RH Ankara) may have influenced the observed differences through strong, nonspecific inflammatory effects rather than typical chronic infection mechanisms. The result of Ihara et al. (2016) showed also a progressive decrease of serotonin in rodent amygdala (221), but the dopamine level in amygdala was fluctuating. The decrease was noted 40 days post infection (dpi), but after 52 dpi there were no longer any significant differences (221). The authors suggested that although *T. gondii* spreads throughout the brain without any clear preference for specific areas, cortical damage and impairment of neuronal function in the cortex and amygdala, rather than the location of the cysts themselves, appear to be more important for behavioral changes (221). Goodwin et al. (2012), however, noted no significant changes in neurotransmitter, including dopamine levels in prefrontal cortex and striatum in prenatal infected mice (229). The inconsistency among various studies may be associated with the use distinct infection models (congenital, acute, chronic), *T. gondii* strain showing distinct virulence, as well as inoculation dose, which Goodwin et al. addressed in their discussion (229). In fact, it is not only the changes in the neurotransmitter levels that are not fully understood; the mechanisms underlying these changes are also elusive. DBH is an enzyme obligatory to convert dopamine into noradrenaline (**Figure 2**) and its suppression leads to noradrenaline level reduction (233). Indeed, DBH activity was found to be reduced during *T. gondii* infection, but exclusively among male rodents, as it has been proved to correlate with the levels of estrogen receptor ESR1 (230). Similarly, in another study altered short-term memory was observed only in male mice (229). Interestingly, both sexes of infected *Dbh^-/-^* mice showed lower number of activated neurons in brain regions responsible for defensive reaction (229). Moreover, it was shown that pharmacological inhibition of DBH along with AR inhibition results in decreased defensive reactions, what confirms the role of noradrenaline during the course of *T. gondii* infection (231). Besides adrenaline and noradrenaline, the parasite may also affect the signaling of their precursor – dopamine (234). The provoked increase of dopaminergic activity and altered neurotransmitter biosynthesis pathways may result from *T. gondii* expression of the genes encoding tyrosine and phenylalanine hydroxylases, involved in the conversion of phenylalanine into tyrosine, which is further converted into L-DOPA – a dopamine precursor (**Figure 2**) (234). This was confirmed by both *in vitro* and *in vivo* experiments, which revealed that *T. gondii* cysts expressing tyrosine hydroxylase increase dopamine levels in infected neurons (218). Additionally, the recent study indicated that *Toxoplasma* promotes neurons to release extravesicles and lncRNA, which interfere with noradrenergic pathways (235). These extravesicles most likely impact the neurophysiology by spreading among non-infected adjacent cells and transferring lncRNA to downregulate DBH gene expression, modify the structure of chromatin (235), and may lead to sequential decrease of noradrenaline and neuroinflammation enhancement. Interestingly, the induced inflammatory response against *T. gondii* driven by TLR11/TLR12 dimer followed by the release of IL-12 (236, 237) promotes the production of IFN-γ by activated NK and T cells (238, 239), which is known to cause tissue damage (240). IFN-γ reduces phenylalanine and tyrosine levels via guanosine triphosphate cyclohydroxylase 1 activation (241), possibly leading to decreased levels of catecholamines. Despite complicated interactions between *T. gondii* and immune/biochemical mechanisms regulating adrenergic pathways, the overall effect shows reproducible pattern. Patients suffering from toxoplasmosis show elevated noradrenaline and adrenaline levels compared to healthy individuals (214). It was also demonstrated that *T. gondii* upregulates the expression and the release of TGF-β (242, 243). Both adrenaline/noradrenaline and TGF-β exhibit anti-inflammatory activity, and the elevated levels of all three molecules may reflect a modulatory effect of the protozoan aimed at inducing immunosuppression (244, 245). However, it is not entirely clear whether the alterations of neurotransmitter release are mediated by *T. gondii* or result from the direct action of the immune system to regulate its activity. These facts may lead to contradictory conclusions and the current state of art does not allow to clearly conclude whether the changes have positive or negative effect on parasite’s survival. Nevertheless, recent experiments with guanabenz provide us with new insights. Guanabenz is α_2_-AR agonist showing antiparasitic effect (246). It reduces parasite burden in the brain and reverses hyperactivity in BALB/c and C57BL/6 mice. Its administration contributes to reduced neuroinflammation in BALB/c mice (247), and dampened release of pro-inflammatory cytokines GM-CSF, IL-6, IL-1β, and TNF by macrophages (248). Therefore, since guanabenz mechanism of action is very similar to natural neurotransmitters like noradrenaline (249), it can be suggested that neurotransmitter modulation mediated by *T. gondii*, and sequential direct pro-inflammatory and/or anti-inflammatory responses induction may partially favor both parasite latency and persistence. Interestingly, AR agonists may also affect the parasite directly. It was observed that clonidine (α_2_-AR agonist) decreases the number of *T. gondii*-infected macrophages (250). However, it seems that this molecule act directly on the parasite (not on macrophages) since only *T. gondii* pretreatment with clonidine resulted in the reduction of infected cells, while macrophage pretreatment had no effect on infection (250). The non-coherent and sometimes contradictory observations may indicate that various AR agonists may interfere with other, not yet defined, biological pathways and affect both parasite and immune cell activity. It is also worth noting that many of the pharmacological agents used (*e.g.* clonidine, guanabenz) exhibit pleiotropic and off-target activities, which complicates the interpretation of these findings as strictly adrenergic. Therefore, the contribution of adrenergic signaling to the observed effects should be interpreted with caution.

#### 3.1.4 Leishmania spp.

Another protozoan-borne disease causing substantial morbidity among humans is leishmaniasis. It is caused by an intracellular protozoan parasite, *Leishmania* spp., which is transmitted by sandflies between mammalian hosts (251). Humans may suffer from infection spread by at least 20 species of the parasite, including *L. donovani*, *L. mexicana*, *L. tropica*, *L. major*, and *L. aethiopica* (252, 253). The disease generally manifests itself in three clinical forms: cutaneous, mucocutaneous and the most severe visceral leishmaniasis, and the symptoms’ exacerbation depends also on the particular parasite species (254). Upon a sandfly bite, *Leishmania* passes through the skin, enters macrophages, multiplies and disseminates to other macrophages or other cells in various tissues (255). Cutaneous leishmaniasis is often recognized by one or multiple cutaneous lesions at the bite site. In contrast, visceral leishmaniasis is characterized by different non-specific symptoms such as fever, weight loss, and enlarged lymph nodes; patients also usually exhibit an enlarged spleen and liver (253, 256). Given the increased attention to neuroinflammation in the context of infections, we hypothesize that adrenergic signaling may be involved in neuroimmune crosstalk during *Leishmania* infection. Although there are limited data regarding the involvement of adrenergic pathways in *Leishmania* infection, some studies provide intriguing premises suggesting a connection between the infection and the changes in neurotransmitter release. An *in vitro* study demonstrated that adrenaline and noradrenaline inhibit parasite attachment to hamster macrophages (257). Pre-treatment of macrophages, parasites, or both resulted in the same inhibitory effect at concentrations of 10–^4^ and 10–^5^ M for both catecholamines (257). Similarly, Kar et al. (2018) observed a reduced growth of the parasite in a culture treated with adrenaline, isoproterenol, and propranolol (258). However, these *in vitro* studies may not reflect the interactions occurring *in vivo*. Moreover, the concentrations of compounds used in these studies must be carefully considered, as the results could arise from the use of non-physiological doses.

Catecholamines and other hormones are molecules strongly associated to stress (259); therefore, the potential interaction between *Leishmania* and catecholamines could be investigated in stressed subjects. A study by Ruiz et al. (2003) showed that stress increases mouse susceptibility to *L. donovani* infection as well as exacerbates the existing symptoms (260). Given that stress correlates with higher levels of catecholamines (261, 262), we hypothesize that infection progression is directly influenced by these compounds. However, stress is also mediated by glucocorticoids, cytokines, and other mediators’ release, which, in some cases, may have redundant effects (263). Thus, catecholamines cannot be considered the only factor impacting the results. To address this complexity, the role of ARs during the infection was investigated with the use of AR blockers (264). Propranolol, a non-selective β-AR antagonist, reduces footpad thickness and parasite load in mice infected with *L. mexicana* LV4. It also enhances the proliferation of CD4^+^ and CD8^+^ T cells, which release IFN-γ (264). Similarly, mice infected with *L. major* exposed to β_1_-AR antagonist, atenolol, show reduced hyperalgesia and TNF-α level, which is known to be associated with pain sensation (265). These studies confirm the above-mentioned *in vitro* data that adrenergic signaling plays a role in the progression of *Leishmania* infections. This encourages the consideration of the use of AR antagonists for the control of leishmaniasis. Nevertheless, further research is required to expand the current understanding of this topic.

### 3.2 Helminths

**Figure 4:**
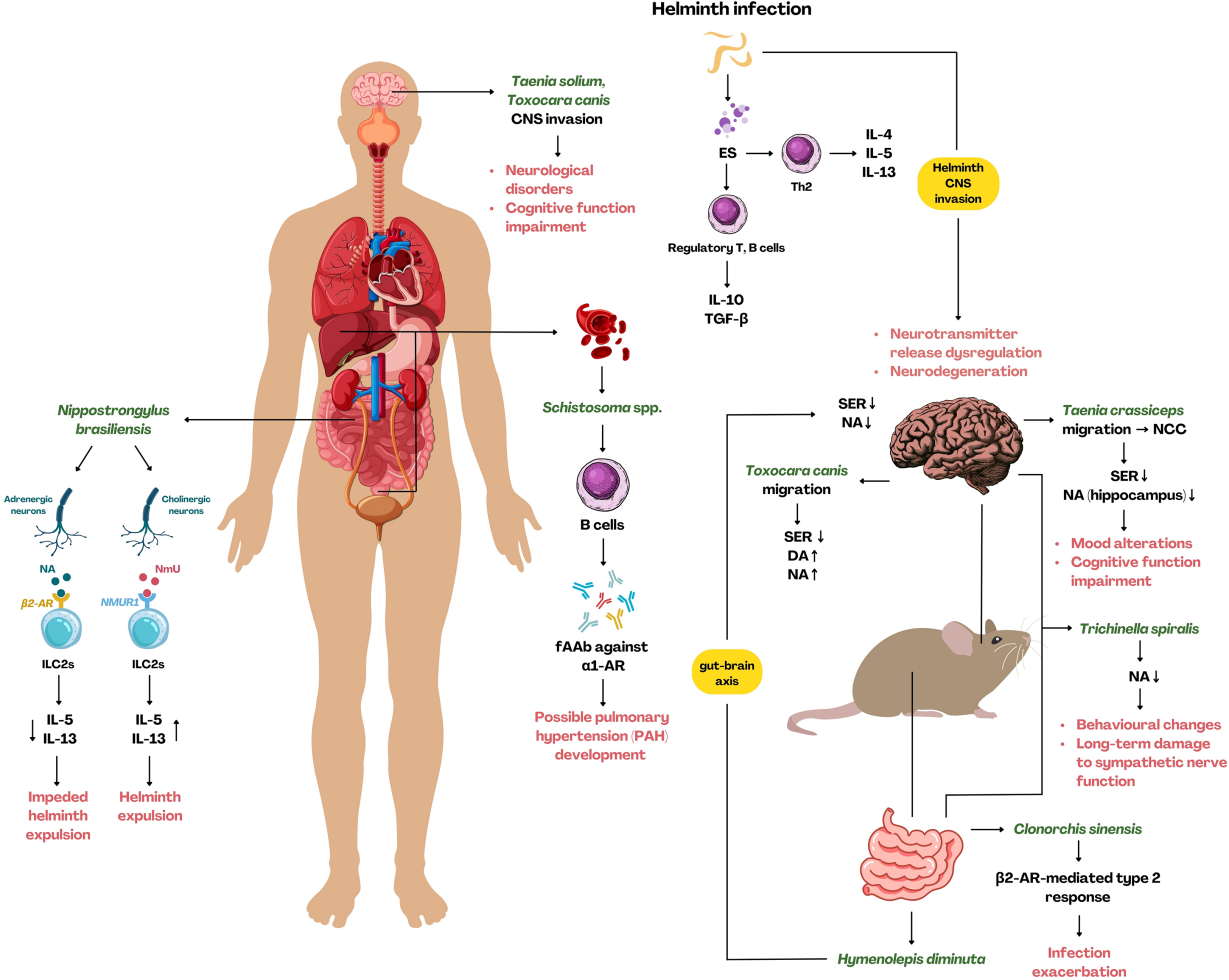
The impact of helminth infection on the immune system, adrenergic receptors, and neurotransmitters. Helminth-derived ES products lead to Th2-mediated response resulting in IL-4, IL-5, and IL-13 release. Simultaneously, ES raise an anti-inflammatory response mediated by regulatory T/B cells. *Nippostrongylus brasiliensis* affects neurons in the intestine, causing noradrenaline or neuromedin U release, leading to contradictory secretion of IL-5 and IL-13 by ILC2s – increased IL-5 and IL-13 facilitate helminth expulsion, while reduced IL-5 and IL-13 caused by NmU binding provides the opposite effect. *Taenia* spp. and *Toxocara* spp. opportunistic CNS invasion in humans and mice causes alterations in neurotransmitter release, followed by various neurological symptoms, including cognitive function impairment and behavioral changes. Neuron function impairment and behavioral changes are observed during *Trichinella* sp*iralis* infection, either in the intestinal or CNS. *Hymenolepis diminuta* intestinal infection causes a decrease in serotonin and noradrenaline levels in the brain, indicating the interplay between gut and brain. *Schistosoma* spp. infection activates plasma cells to produce fAAbs promoting PAH development and infection exacerbation. *Clonorchis sinensis* infection exacerbation, manifested by liver fibrosis and hepatobiliary damage, is possibly the result of β_2_-AR signaling mediating the induction of M2 macrophages. AR, adrenoreceptor; ILC2s, innate lymphoid cells type 2; CNS, central nervous system; fAAb, functional autoantibodies; ES, excretory-secretory products; SER, serotonin; NA, noradrenaline; DA, dopamine; NCC, neurocysticercosis; PAH, pulmonary arterial hypertension. [Created using Canva Pro (licensed version) based on literature discussed in this work].

Helminths are an artificial group of multicellular invertebrates commonly referred to as parasitic worms. They are generally classified into three categories: nematodes, cestodes, and trematodes (266). Helminths have long, flat, or round bodies and are a leading cause of mortality in developing countries due to their high prevalence (267, 268). They usually have complex life cycles with a number of developmental stages: egg, a variable number of larval stages, and finally adult form; this complexity raises challenges for both diagnosis and treatment of the infections (266). During the course of evolution, helminths have developed unique abilities to modulate the host immune system, enabling their reproduction and survival (7). They trick the host immune system through shedding various antigens between developmental stages and release immunomodulatory antigens directly impacting the host immune system (7, 269, 270). Moreover, unlike protozoa, worms cannot be easily phagocytized, primarily due to their size and motility (271). Helminth infection is generally associated with type 2 and regulatory responses (272–275). Th2 response is triggered by Th2 cells activation, IL-4, IL-5, IL-13 release, as well as eosinophil promotion, and subsequent IgE production (272, 276–278), whereas Treg cells shift the response toward immunosuppressive phenotype through IL-10 and TGF-β cytokines, which may be induced directly by the helminth or through the microbiome (7, 279, 280). All those helminth-induced effects are, in the majority, associated with the antigens actively released – ES – or shed from a tegument by the worms (Surface Antigens – SA) (7). These molecules may impact the activation of CD4^+^ Tregs (281), CD8^+^ Tregs (282), and regulatory B cells (283), as well as the production of TGF-β, IL-10, and Th2 cytokines (284, 285). Therefore, due to their significant roles during the infections, they have been identified as potential factors useful in mitigating autoimmune and allergic diseases’ symptoms (286). Unfortunately, despite well-established knowledge regarding the interplay between immune system and host adrenergic signaling, the literature data exploring this crosstalk during helminth infections are limited. The following sections highlight the challenges that warrant further investigation of this topic in the context of cestodes, nematodes, and flukes. The graphic summary of this chapter is presented in **Figure 4**.

#### 3.2.1 Cestodes

Flatworms such as *Taenia solium* and *Taenia crassiceps* may occupy the CNS, causing neurocysticercosis (NCC), which can be manifested by either severe neurological symptoms or no clinical signs (287), with the latter associated with worms’ ability to modulate the immune system. A powerful immunomodulatory fraction is constituted by ES products released by the parasite. They are composed of numerous molecules that usually shift the response toward an anti-inflammatory phenotype, facilitate immune response evasion, and favor parasite survival and spreading (7). *Taenia* spp. possibly increases the secretion of prostaglandin E2 (PGE_2_) (288), somatostatin (289), and neuropeptide substance P (290) enhancing infection symptoms and promote granuloma formation. Moreover, cestodes ES prevent the activation of DCs (291) and microglia (292), and on the other hand induce alternatively activated macrophages (AAM) (293), Tregs (294), and plasma cells (295). It is directly associated with preventing Th1 response (296), engaging IL-10 and TGF-β in the CNS response (297), and therefore preventing the recruitment of leukocytes to CNS (298, 299). All these actions are to create a comfortable niche for the parasite, facilitating its survival and evasion from the immune response. Unfortunately, our comprehension of the crosstalk between the immune and nervous system during *Taenia* spp. infection is very superficial. Nevertheless, the analyses and combination of literature data regarding the immune response and nervous system in the context of NCC indicate tight cooperation of these systems (300–302). This interaction is associated not only with the site of the infection but also shows systemic outcomes and impacts the release of neuromediators observed beyond the tissue occupied by the larvae. This was proved by Morales-Montor et al. (2014), who showed the decreased levels of serotonin (in *T. crassiceps*-infected female mice) and increased levels of noradrenaline in the hippocampus (in *T. crassiceps*-infected male mice) (303), even though the infection was intraperitoneal, and no larvae were found in the brain. Infected mice also showed altered mood and short-term memory disturbances (303). Similar effects were observed during gastrointestinal infection with *Hymenolepis diminuta* (304). The infection in rats showed improvement in spatial memory (water maze test) and recognition of new objects (NOR test), which was likely correlated with lower levels of noradrenaline in the prefrontal cortex, cerebellum, and striatum and decreased serotonin level in the striatum (304). This might at least partially explain the occurrence of neuropsychiatric symptoms and cognitive function impairment in humans due to *T. solium* induced NCC (305). Although these results only partially explore the role of adrenergic signaling, they show a premise for further investigation.

#### 3.2.2 Nematodes

Similar to some cestodes, nematode larvae are also capable of active migration and reaching the CNS, intestine, and other organs, causing inflammation and various clinical symptoms (306, 307). Nevertheless, the knowledge regarding nematode impact on host signalization through catecholamines is still in its infancy. Despite a small amount of data regarding nematode interplay with host adrenergic signaling, the epidemiological significance of gastrointestinal nematodes led to various research focusing on parasite-host immune interaction, which gives some insights into parasite-hormone crosstalk. ILCs are derived from common lymphoid progenitors and have been described as the TCR-deprived counterparts of the T cells (308). They are one of the first cells to orchestrate the rise of the immune system response against the invaders (309), with ILC2s being one the most prominent population during parasitic infection in the intestine (310). ILC2s express neuromedin U receptor 1 (NMUR1) and β_2_-AR, which enable direct interaction with neurons (31, 311), as they release neuromedin U (cholinergic neurons) and noradrenaline (adrenergic neurons) (31, 312). Neuromedin U promotes ILC2s to release prominent Th2 cytokines (IL-5, IL-13), which further mediate host defense against intestinal nematode – *N. brasiliensis* (312, 313). In contrast, noradrenaline seems to have the opposite effect; upon binding to β_2_-AR on ILC2s, it diminishes IL-5 and IL-13, subsequently impeding type 2 response against *N. brasiliensis* and its expulsion (31). A recent study demonstrated that in mice with ILC2 deficiency *N. brasiliensis* infection alters the intestinal transcription profile, as shown by RNA-Seq analysis, and impairs the appropriate inflammatory reaction to the parasite, highlighting the essential role of ILC2s in host defense (314). Although changes in gut functioning are rapidly transmitted to the brain through the vagus nerve (315) and result in mood changes (316, 317), the role of such signaling and its adrenergic component during nematode infections is still to be determined. Nematodes may also settle in the brain, and almost 30 years ago, Abdel Ghafar et al. (1996) suggested that neurotransmitter level alterations might be associated with behavioral changes in mice (318). The authors demonstrated the decrease of dopamine or serotonin and noradrenaline levels in the brain during *Toxocara canis* and *Trichinella* sp*iralis* infections, respectively, which was partially improved by mebendazole treatment (318). These results are not surprising since brain-dwelling parasites may impact neural cells locally. Similarly, a muscle-dwelling nematode (*Trichinella* sp*iralis)* infection causes noradrenaline decline in rats’ intestines, myenteric plexi, which persists up to 100 dpi (319).

Some efforts have also focused on deciphering platelets’ roles during nematode infections. Platelets are the smallest blood compounds with a medium life span (7–10 days) (320), and, when activated, they release various molecules, acting as antimicrobial agents, as well as immunomodulators (321). Although they are not a substantial element of adrenaline signaling, they express ARs (322, 323) and might contribute to general outcomes of the response. Their role, however, in abrogating adrenaline signaling is likely of minor significance. In an early study performed over 40 years ago, it was found that *Ancylostoma* infection in humans causes platelet dysfunction, which may be associated with ADP release attenuation (324). This platelet dysfunction resulted in the impairment of collagen-induced aggregation and adrenaline-induced second-phase aggregation. These effects were resolved upon the anthelmintic treatment (324), indicating direct parasite impact. Moreover, the dysfunction is likely to be ES dependent. The platelet inhibitor protein (HPI), released by the worm binds to α2β1 and αIIbβ3 integrins leading to declined platelet adhesion to fibrinogen and collagen, followed by impaired adrenaline-, thrombin-, and ADP-induced aggregation (325). On the other hand, *Dirofilaria immitis* ES are likely to promote platelet activation. Platelets obtained from infected dogs incubated with heartworms exhibited increased adrenaline- and ADP-induced aggregation (326). In platelets from dogs with occult heartworm disease, increased platelet activation was observed, what indicates that the increase was not associated with infection severity and microfilariae burden (326). Therefore, it can be assumed that platelet reactivity is mediated through the factors present in plasma, such as parasite-derived antigens. Takashima et al. (2016) confirmed that supplementing the dog platelet-rich plasma with *D. immitis* extract causes rapid and irreversible platelet aggregation in a concentration-dependent manner (327). These findings suggest that platelets may participate in orchestrating the immune response indirectly through stimulating or repressing adrenaline pathways.

Another novel and significant area of research is exploring the potential association of nematode infections and neurodegenerative/neuropsychiatric disorders like cognitive function impairment, schizophrenia, dementia or Parkinson’s disease. Conventionally, nematodes infect CNS in small numbers (328, 329), however these infections may last for months, even years, which was proved on mice and monkeys (330, 331). The data from monkeys allow to hypothesize that similar interactions may occur in human population. Unfortunately, the data regarding adrenergic signaling and neurodegeneration processes during the nematode infection are neither broad nor consistent, at this point. Nevertheless, the presence of nematodes in the CNS may contribute to neurodegeneration, particularly given that the development of neurodegenerative disorders is linked to primary dysregulation of noradrenergic transmission (332, 333) – an alteration also observed during nematode CNS infections (303, 334). Therefore, understanding the molecular mechanisms of interaction between adrenergic signaling and helminths is crucial for establishing critical points in pathogenesis of nematode infections, as well as neurodegenerative diseases, and on this ground – enabling new therapeutic approaches.

#### 3.2.3 Flukes

Despite the huge impact of flukes on public health, to date, there is a paucity of published studies addressing the role of adrenergic signaling in neuroinflammation during trematode infections. Nevertheless, some data are available for blood flukes from *Schistosoma* genus. *S. mansoni* and *S. japonicum* primarily enter the skin and migrate through blood circulation to the superior and inferior mesenteric veins or hemorrhoidal veins (335); sometimes they can migrate to the intestine (336), but also to the CNS, leading to neuroschistosomiasis (337). After the schistosomes mature and pair, they lay eggs that can reach the CNS (338). Generally, *Schistosoma* spp. infection strongly skews the immune response towards the Th2 phenotype and promotes a granulomatous reaction (339–341). Granulomas may also develop around *Schistosoma* spp. eggs present in the CNS and lead to various neurological symptoms (338), such as headache, seizure, and increased intracranial pressure, myelopathy, focal motor deficits, and loss of consciousness (335). The symptoms of the infection are typically associated with the tissue occupied by the fluke, which is subjected to physical contact with the parasite and its ES products (342–344). Flukes exhibit a wide range of modulatory mechanisms, including amplifying Th2-type cytokine release, reducing MHC II expression to disrupt antigen presentation, inducing Treg differentiation, among others, as reviewed elsewhere (345). Patients suffering from *S. mansoni* infection, with developed hepatosplenic clinical form, can develop pulmonary arterial hypertension (PAH), which is a severe, however less frequent, complication (346). The pathogenesis of PAH is partially mediated by adrenergic signaling, through noradrenaline, but also through angiotensin II, endothelin I, TGF-β, and Th1, Th2, Th17-like cell activity (347). Moreover, in recent study, it has been shown that infected patients produce functional autoantibodies (fAABs) against three different GPCRs: the α_1_-AR, the endothelin-1 receptor (ETA1), and the angiotensin II receptor (AT1) (347), which may play a significant role in the development of PAH. In fact, fAABs were proposed to serve as a potential marker for the development of PAH in the later stages of schistosomiasis (347). Another fluke, which was investigated for association with adrenergic pathways, is *Clonorchis sinensis.* Koda et al. (2021) proposed that during the infection, adrenergic signaling through β_2_-AR can contribute to the exacerbation of the clinical signs of the infection (348). Macrophages obtained from *Adrb2*^-/-^ mice infected with *C. sinensis* showed reduction in Th2 cytokine levels as well as mitigated hepatobiliary damage and liver fibrosis (348). This may be especially significant since the development of M2 macrophages, which are key players in fibrosis, depends strongly on β_2_-AR (348). Despite these findings, research on adrenergic pathways and neuroimmune responses in schistosomiasis remains limited, particularly across a broader range of trematode species. This highlights the importance of conducting new studies in this area.

## 4 Conclusions

Adrenergic signaling plays a significant role in shaping immune and physiological responses during parasitic infections. Through ARs, catecholamines influence inflammation, immune cell function, and the severity of tissue damage (245). Protozoan parasites often manipulate these pathways to evade immune defenses and facilitate systemic dissemination, while helminths exploit them to sustain chronic infections.

Although adrenergic signaling in the context of parasitic infections is underexplored, this review opens new significant directions. Firstly, adrenergic signaling is closely linked to mechanisms underlying neurodegenerative diseases, many of which are becoming increasingly prevalent in modern societies (349). The presence or altered distribution of adrenergic receptors, as well as dysregulated levels of their agonists, can reflect ongoing pathological processes and finally point to potential therapeutic strategies. In the context of very common intestinal infections, adrenergic signaling may contribute to parasite persistence by suppressing local immune activity and reducing intestinal motility, thereby impairing parasite expulsion. Understanding how ongoing mechanisms differ depending on host species, location and parasite type could be key to developing precise and safe treatments that act selectively on defined receptor subtypes and tissues, allowing for the restoration of host physiology. An interesting perspective that emerges from current data is the possibility of repurposing existing adrenergic drugs for antiparasitic therapy, which, we believe, deserves greater attention. Agents such as β-AR blockers or α_2_-AR agonists, already characterized in terms of safety and pharmacodynamics, could be rationally adapted to interfere with parasite development or host immune modulation (199). Despite the clear advantages of this strategy, the current research landscape is dominated by the pursuit of novel compounds rather than the exploration of well-known molecules with new therapeutic purposes. The unique capability of immune evasion by parasites has been extensively explored (350). The induction of immune tolerance or suppression facilitates their survival, and understanding these mechanisms may offer valuable insights for controlling autoimmune and allergic diseases. Nevertheless, protozoan and metazoan parasites interfere not only with the immune system but also exert a significant effect on other aspects of an organism’s functioning. Adrenergic signaling is a significant component of homeostasis regulation, and its dysfunction is associated with metabolic, mental, or neurodegenerative disorders (245). Unfortunately, the phenomenon of parasite-adrenergic signaling interplay is much less explored than parasite-immune system interactions. This is due to the fact that neural-immune-biochemical interactions are extremely complicated, and these two aspects have been treated separately. Moreover, restricted resources often do not allow researchers to investigate the immune response, neural biochemistry, and changes in hormone levels simultaneously. Fortunately, nowadays it is possible to combine all the data and search for patterns between these aspects of infections, deciphering the complex reaction of the organism to the intruder. This review indicates the substantial association between parasite infections and adrenergic signaling, which interplay with the immune system, yet it is still difficult to determine the cause and the outcome; in numerous cases, it is not clear if the parasite impacts directly the immune system resulting in changes in adrenergic signaling or if change of the immune response is an outcome of abrogation in adrenergic signaling, or, finally, if the changes in those two aspects are outcomes of other, yet undefined mechanisms. This gap in the knowledge calls for an understanding of these interactions and opens new avenues of research. Knowledge gained from studying how parasites manipulate adrenergic signaling gives perspectives beyond parasitology and the development of novel methods for disease control. Although the use of parasites as a remedy for diseases associated with an exacerbated immune response held great potential, it was intuitive, as these infections and the immune response are directly linked (351, 352). Adrenergic signaling plays a role in metabolic, mental, or neurodegenerative disorders, but also crosswalks with the immune response. Here, the association is not direct and may be more subtle, although significant. Novel technologies, such as AI and computational sciences, create new perspectives for analyzing new data and reanalyzing numerous previously published datasets deposited in various repositories. Previously, this approach was beyond us. The results may lead to the development of new strategies for controlling metabolic, mental, or neurodegenerative disorders, which are now in their infancy.

In conclusion, understanding the molecular details of adrenergic signaling in host–parasite interactions will not only deepen our knowledge of infection biology but may also provide new directions for therapies targeting neurodegenerative, autoimmune, and inflammatory disorders.
